# Total Neoadjuvant Therapy for Rectal Cancer: Why Japan Says “Not Yet”

**DOI:** 10.1002/ags3.70121

**Published:** 2025-11-12

**Authors:** Kay Uehara, Akihisa Matsuda, Takeshi Yamada, Aitsariya Monkhonsupphawan, Hiroshi Yoshida

**Affiliations:** ^1^ Department of Gastroenterological Surgery Nippon Medical School Tokyo Japan; ^2^ Department of Surgery, Siriraj Hospital Mahidol University Bangkok Thailand

**Keywords:** locally advanced rectal cancer, non‐operative management, total neoadjuvant therapy

## Abstract

Total neoadjuvant therapy (TNT) has rapidly gained global acceptance as a standard treatment for locally advanced rectal cancer (LARC). Supported by multiple phase III trials, TNT improves pathological complete response (pCR) rates, enhances systemic control, and expands opportunities for non‐operative management (NOM). These advantages have led to its inclusion in major international guidelines as a core strategy for stage II/III rectal cancer. However, not all regions have embraced TNT. Japan's 2024 colorectal cancer treatment guidelines weakly recommend against the routine use of TNT or NOM—making it one of the few countries to diverge from the global trend. This stance does not reflect a rejection of evidence, but rather the realities of a healthcare system where rectal cancer is often treated in non‐specialized institutions. Unlike consensus guidelines designed for subspecialists, the Japanese guidelines are tailored to general surgeons practicing in a wide range of settings, many of whom manage rectal cancer infrequently. This reflects a broader challenge of limited centralization within Japan's otherwise equitable healthcare system. TNT also presents unresolved concerns, including toxicity, uncertain survival benefit, and increased surgical complexity—issues particularly relevant in resource‐diverse environments. This review examines the global evolution of TNT and Japan's restrained response, analyzing key trials, guideline positions, and barriers to implementation. Japan's approach reflects practical realities rather than opposition, emphasizing the need to tailor TNT to each country's healthcare setting. The future of TNT lies not in universal application, but in thoughtful integration that balances oncologic efficacy with local context and patient‐centered care.

## Abstract 2

Total neoadjuvant therapy (TNT) improves tumor regression, systemic control, and feasibility of non‐operative management, and is now endorsed by major international guidelines for locally advanced rectal cancer. However, Japan's 2024 guidelines weakly recommend against routine TNT or NOM, reflecting healthcare system constraints, surgical practice patterns, and limited domestic evidence. This review analyzes global and Japanese perspectives, highlighting the need for context‐specific trials and tailored implementation.

## 1 Introduction

The management of locally advanced rectal cancer (LARC) has evolved considerably over recent decades. The adoption of total mesorectal excision (TME), followed by preoperative radiotherapy, significantly improved local control [1, 2, 3, 4]. More recently, total neoadjuvant therapy (TNT)—the delivery of both radiotherapy and full‐course systemic chemotherapy before surgery—has gained increasing global attention [5, 6, 7, 8, 9, 10, 11, 12, 13, 14, 15].

TNT has been associated with higher pathological complete response (pCR) rates [6, 8, 10], better control of distant metastases [7, 8, 10], and increased feasibility of non‐operative management (NOM) strategies [14, 15]. These advantages have led major international guidelines—including those from the NCCN, ESMO, and the French Intergroup—to recommend TNT for patients with high‐risk LARC [16, 17, 18].

In contrast, Japan has remained cautious. The 2024 edition of the Japanese colorectal cancer treatment guidelines marked the first formal mention of TNT, concluding with a weak recommendation against its routine use (currently available only in Japanese; an English translation is in preparation and not yet officially published or available for citation). This conservative stance reflects several Japan‐specific factors, including skepticism regarding the survival benefit of TNT [6, 19], a historical preference for upfront surgery as the standard of care, and the available alternative treatment of lateral pelvic lymph node dissection (LPLND) to improve lateral local control [20].

This review aims to critically examine global and Japanese perspectives on TNT, synthesize current evidence, and discuss challenges and opportunities for its context‐specific application in Japan. A literature search was conducted in PubMed, Embase, Cochrane Library, and journal publisher websites through May 2025, using the terms “locally advanced rectal cancer,” “total neoadjuvant therapy,” “non‐operative management,” “induction,” and “consolidation.”

## 2 Global Momentum: Clinical Trials

The rationale for TNT in LARC stems from the limitations of conventional chemoradiotherapy (CRT), which, while effective for local control, has not substantially improved long‐term survival [2, 3, 4]. By delivering full‐dose systemic chemotherapy prior to surgery, TNT aims to enhance systemic control without compromising local outcomes. Over the past decade, some large randomized trials (RCTs) have explored this approach and collectively reshaped global treatment standards [5, 6, 7, 8, 9, 10, 11, 12, 13, 14, 15].

### 2.1 Comparative Evidence—TNT Vs. Conventional CRT

Among key randomized clinical trials (RCTs) (Table 1), the RAPIDO study compared short‐course radiotherapy (SCRT) followed by chemotherapy versus conventional CRT. TNT improved pCR (28% vs. 14%, *p* < 0.001) and lowered 3‐year distant metastasis (20.0% vs. 26.8%, *p* = 0.005), though overall survival (OS) was not significantly different, and locoregional recurrence was higher in the TNT group (10% vs. 6%, *p* = 0.027) [7, 21].

**TABLE 1:** Key outcomes from landmark Phase iii trials of TNT in locally advanced rectal cancer.

Trial (Enrolment period)	Arm/strategy	*n*	R0 resection	*p*	pCR	*p*	pCR + cCR	*p*	LR	*p*	DM	*p*	DFS	*p*	OS	*p*
Polish II (2008–2014) [5, 6]	TNT: SCRT → FOLFOX → TME	261	77%	0.07	16%	0.17	—		35% (8‐yr)	0.6	36% (8‐yr)	0.54	43% (8‐yr)	0.65	49% (8‐yr)	N.S.
	CRT → TME	254	71%		12%				32% (8‐yr)		34% (8‐yr)		41% (8‐yr)		49% (8‐yr)	
RAPIDO (2011–2016) [7, 19, 21]	TNT: SCRT → FOLFOX/CAPOX → TME	462	90%	0.87	**28**	**< 0.0001**	—		**10.2% (5‐yr)**	**0.027**	**23% (5‐yr)**	**0.011**	**28% (5‐yr)** ^a^	**0.048**	82% (5‐yr)	0.5
	CRT → TME	450	90%		**14**				**6.1% (5‐yr)**		**30% (5‐yr)**		**34% (5‐yr)** ^a^		80% (5‐yr)	
PRODIGE‐23 (2012–2017) [8, 9]	TNT: FOLFIRINOX → CRT → TME	231	95% (201/211)	0.63	**28**	**< 0.0001**	—		5.3% (7‐yr)	N/R	20.7% (7‐yr)	0.066	**67.6% (7‐yr)**	***p* = 0.048**	**81.9% (7‐yr)**	**0.033**
	CRT → TME	230	94% (202/214)		**12**				8.1% (7‐yr)		27.7% (7‐yr)		**62.5% (7‐yr)**		**76.1% (7‐yr)**	
STELLAR (2015–2018) [10]	TNT: SCRT → CAPOX → TME	298	91.5% (215/235)	0.198	16.6%	N/R	**21.8% (3‐yr)**	**0.002**	8.4% (3‐yr)	0.461	77.1% (3‐yr)^b^	0.475	64.5% (3‐yr)	Noninferiority *p* < 0.001	**86.5% (3‐yr)**	**0.033**
	CRT → TME	293	87.8% (202/230)		11.8%		**12.3% (3‐yr)**		11.0% (3‐yr)		75.3% (3‐yr)^b^		62.3% (3‐yr)		**75.1% (3‐yr)**	

*Note:* Bold text indicates items with significant differences.

Abbreviations: cCR, clinical complete response; CRT, chemoradiotherapy; DFS, disease‐free survival rate; DM, distant metastasis rate; iCR, incomplete clinical response; LR, local recurrence rate; nCR, near‐complete response; NOM, non‐operative management; N/R, not reported; N.S., not significant; OS, overall survival rate; pCR, pathological complete response; TME, total mesorectal excision; TNT, total neoadjuvant therapy.

^a^ Disease‐related treatment failure.

^b^ Distant metastasis‐free survival.

PRODIGE‐23 intensified TNT with mFOLFIRINOX prior to CRT, followed by surgery and adjuvant therapy. It demonstrated better pCR and disease‐free survival (DFS), and long‐term follow‐up confirmed a 7‐year OS benefit (81.9% vs. 76.1%, *p* = 0.033) [8, 9]. However, toxicity and concerns over overtreatment remain, especially considering prior data questioning irinotecan's survival benefit in colon cancer [22, 23].

The STELLAR trial from China used SCRT followed by CAPOX and confirmed TNT's non‐inferiority for DFS (64.5% vs. 62.3%, HR = 0.883) with better OS (86.5% vs. 75.1%, *p* = 0.032) [10]. Yet nearly half the patients had low‐risk (cT3N0) disease, making generalization difficult. The absence of DFS superiority raises questions about the clinical value of added intensity in such populations.

### 2.2 TNT Protocols: Induction Versus Consolidation

Recent trials have also examined TNT sequencing—whether chemotherapy should precede or follow radiotherapy. Induction aims to control micrometastases early; consolidation may enhance tumor regression and cCR, key for NOM (Table 2).

**TABLE 2:** Comparative outcomes of induction versus consolidation TNT strategies in key randomized trials for locally advanced rectal cancer.

Trial (Enrolment period)	Arm/strategy	*n*	R0 resection	pCR	pCR + cCR	cCR + nCR (at restaging after TNT)	NOM population (cCR + nCR + iCR)	Regrowth	*p*	TME‐free survival	*p*	LR	*p*	DM	*p*	DFS	*p*	OS	*p*
CAO/ARO/AIO‐12 (2015–2018) [11, 12, 13]	Induction: FOLFOX → CRT → TME	156	92% (130/142)	17%	21% (5‐yr)	—	—	—		—		6.9% (5‐yr)	0.69	21.7% (5‐yr)	0.75	73% (3‐yr)	0.82	85.8% (5‐yr)	0.36
	Consolidation: CRT → FOLFOX → TME	150	90% (128/142)	25%	27% (5‐yr)							5.9% (5‐yr)		23.8% (5‐yr)		73% (3‐yr)		84.2% (5‐yr)	
OPRA (2014–2020) [14, 15]	Induction: FOLFOX/CAPOX → CRT → NOM/TME	158	91% (67/74)	—	—	69% (101/146)	72% (105/146)	**44% (5‐yr) (46/105)**	**0.02**	**39% (5‐yr)**	**0.012**	94% (5‐yr)^a^	0.94	80% (5‐yr)^b^	0.61	71% (5‐yr)	0.68	88% (5‐yr)	0.25
	Consolidation: CRT → FOLFOX/CAPOX → NOM/TME	166	88% (53/60)			73% (116/158)	76% (120/158)	**29% (5‐yr) (35/120)**		**54% (5‐yr)**		90% (5‐yr)^a^		78% (5‐yr)^b^		69% (5‐yr)		85% (5‐yr)	

*Note:* Bold text indicates items with significant differences.

Abbreviations: cCR, clinical complete response; CRT, chemoradiotherapy; DFS, disease‐free survival rate; DM, distant metastasis rate; iCR, incomplete clinical response; LR, local recurrence rate; nCR, near‐complete response; NOM, non‐operative management; OS, overall survival rate; pCR, pathological complete response; TME, total mesorectal excision; TNT, total neoadjuvant therapy.

^a^ Local recurrence‐free survival.

^b^ Distant metastasis‐free survival.

The CAO/ARO/AIO‐12 trial (phase II, Germany) compared these strategies, both followed by surgery. The consolidation arm had a higher pCR (25% vs. 17%) with similar DFS and OS [11, 12, 13]. Though not designed to compare pCR or assess NOM, it supported consolidation's tumor regression advantage.

OPRA (phase II, USA) evaluated sequencing in patients eligible for NOM. At 5 years, the consolidation group achieved higher organ preservation (54% vs. 39%, *p* = 0.012) and less local regrowth, without compromising DFS or OS [14, 15]. This supports consolidation when NOM is a goal.

French trials like GRECCAR14 and NORAD01‐GRECCAR16 now explore biologically adapted induction TNT. GRECCAR14 uses MRI after mFOLFIRINOX to determine whether patients can omit CRT [24]. NORAD01 tests mFOLFIRINOX alone versus CRT [25]. These studies prioritize response‐based tailoring and may be particularly relevant for Japan, where CRT is not uniformly adopted.

## 3 The Promise of TNT: Evidence‐Based Benefits

### 3.1 Achieving pCR: Gateway to Organ Preservation in TNT

pCR—defined as the absence of residual tumor cells in the resected specimen—is a widely reported outcome in neoadjuvant treatment for rectal cancer. A favorable pCR rate is generally regarded as a marker of effective tumor regression, and several studies have demonstrated that patients achieving pCR experience significantly better long‐term outcomes, including DFS and OS, compared with those who do not [26, 27, 28]. Nonetheless, the prognostic value of pCR as a surrogate for survival remains debated. Some meta‐analyses have questioned its reliability in clinical trials, especially regarding its ability to predict long‐term systemic control [29, 30]. Moreover, recent real‐world data suggest that patients who achieve pCR after TNT may have worse survival than those who achieve pCR following CRT alone. This discrepancy may be due to differences in treatment sequencing [31].

Despite these concerns, pCR remains clinically meaningful—especially in the context of organ‐preserving strategies. TNT protocols, particularly those employing consolidation sequencing, have been associated with higher pCR rates than conventional CRT, fueling interest in selective surgical omission [7, 10, 11, 14].

### 3.2 Facilitating Organ Preservation: Expanding the Role of NOM

NOM strategies for rectal cancer were initially explored in the CRT era [32], with cCR observed in about 8%–26% of cases [27]. TNT has expanded the feasibility and reliability of NOM by increasing tumor regression and pCR rates.

A recent large‐scale international real‐world study involving 1585 patients across 61 centers, treated off‐trial with TNT for stage II/III rectal cancer, reported a watch‐and‐wait (W&W) rate of 12.1% and a cCR/pCR rate of 23.2% [33]. These findings underscore the realistic potential for NOM uptake outside controlled trials.

Even in cases of local regrowth, prompt salvage surgery can offer favorable oncologic outcomes, including high rates of R0 resection [15]. NOM reflects a shift toward personalized care, balancing oncologic control with quality of life. However, its success depends on rigorous surveillance and multidisciplinary coordination, often limited to high‐volume centers.

### 3.3 Reducing Distant Failure: TNT'S Systemic Advantage

The primary rationale for TNT is to improve systemic control by delivering intensive chemotherapy before surgery—when micrometastases may still be eradicable.

This rationale has been supported by several phase III RCTs. The RAPIDO study reported significantly lower 5‐year distant metastasis rates with TNT vs. CRT (23% vs. 30%, *p* = 0.011), including fewer liver metastases (9% vs. 15%, *p* = 0.002) [19]. Similarly, PRODIGE‐23 showed better 3‐year disease‐free and metastasis‐free survival, with follow‐up confirming durable oncologic benefit [8, 9].

Importantly, real‐world evidence also supports these findings. In a large international cohort of 1585 patients treated off‐protocol with TNT, the 3‐year event‐free survival (EFS) was 68%, and the 5‐year OS was 79%—comparable to outcomes reported in randomized trials [33].

Together, these data suggest that early systemic chemotherapy in TNT reduces distant relapse and, in some cases, improves survival—making TNT a promising strategy for high‐risk LARC, where distant failure remains the main barrier to cure.

## 4 Pitfalls and Concerns: Unresolved Issues

### 4.1 Uncertain Impact on Long‐Term Survival

Despite compelling evidence that TNT increases pCR rates and reduces distant metastases, its effect on overall survival remains controversial. While some trials, such as the STELLAR and extended follow‐up of PRODIGE‐23, have demonstrated modest yet statistically significant OS improvements [9, 10], others have not shown a survival advantage despite enhanced systemic control.

One possible explanation is the “Association of Treatment‐Related Effects with Shorter Survival” (ATRESS) phenomenon, which suggests that TNT, although effective in tumor downstaging, may inadvertently select for resistant subclones, delay definitive surgery in non‐responders, or increase treatment‐related morbidity. These factors could counteract the long‐term benefits of early systemic chemotherapy [34].

Supporting this concern, an updated analysis of the RAPIDO trial reported that patients in the TNT group had significantly worse survival after the onset of distant metastasis compared to those in the standard‐care group (median 2.6 vs. 3.2 years; HR = 1.39, *p* = 0.04). This paradox may reflect reduced effectiveness of post‐metastasis treatments in patients who had already received intensive neoadjuvant chemotherapy, thereby limiting further therapeutic options.

### 4.2 Local Control Concerns

Although TNT improves tumor regression and distant control, several studies raise concerns about its potential to compromise surgical safety and local disease control [21, 35, 36, 37, 38]. Notably, the RAPIDO trial reported a significantly higher rate of locoregional recurrence after surgery in the TNT group compared to CRT (10% vs. 6%, *p* = 0.027) [21]. This unexpected finding has led to questions about possible oncologic trade‐offs—particularly with SCRT‐based TNT followed by delayed surgery.

Recent analyses have suggested that this excess risk may partly reflect treatment‐induced tumor downsizing, which can mask microscopic deposits or residual tumor cells [21, 35]. In this context, preoperative surgical planning should be based primarily on baseline MRI findings rather than on post‐treatment regression, in order to avoid underestimating the true extent of disease. This principle is particularly relevant in centers adopting organ‐preserving or sphincter‐preserving approaches.

While RAPIDO reported a higher local recurrence rate in the TNT group, this finding was not consistently observed in other major TNT trials. In the STELLAR trial, local recurrence rates at 3 years were virtually identical between the SCRT‐based TNT group and the conventional CRT group (6.1% vs. 5.9%, respectively), indicating that SCRT‐TNT may not inherently compromise local control [10]. Similarly, in the Polish II trial—which evaluated fixed cT3/cT4 tumors—the 8‐year cumulative incidence of local failure did not differ significantly between the SCRT plus consolidation chemotherapy arm and the conventional CRT arm (35% vs. 32%; HR = 1.08, *p* = 0.60) [6].

Furthermore, regimen sequencing appears to impact the local recurrence rate. A comparative analysis of the CAO/ARO/AIO‐04 and the CAO/ARO/AIO‐12 trials demonstrated a lower local recurrence rate with consolidation TNT (3.6%) compared to induction TNT (6.2%) following conventional CRT, suggesting a potential benefit of the consolidation strategy in preserving local control [39].

Taken together, these data highlight that local recurrence rate outcomes vary significantly depending on treatment schedule, timing, and institutional experience. This heterogeneity underscores the importance of surgical learning curves and suggests that SCRT‐based TNT should not be dismissed solely based on RAPIDO findings, especially in high‐volume centers with experience in delayed surgery following radiation.

Regimen‐specific findings from real‐world data provide important nuance. In the large cohort (*n* = 1585), patients receiving a PRODIGE‐23–like regimen experienced a serious adverse event (SAE) rate of 23.5%, but had lower rates of local progression at treatment failure compared to other regimens: 3.9% (PRODIGE‐like) vs. 8.9% (RAPIDO‐like), 11.9% (OPRA induction‐like), and 10.1% (OPRA consolidation‐like). Distant progression rates were similar across regimens [33]. In unadjusted analyses, EFS and OS were also longer in the PRODIGE‐23–like group. However, after propensity matching, no statistically significant differences were observed between regimens for any oncologic outcome (EFS, OS, local/distant progression). SAEs were most frequently reported in patients receiving a RAPIDO‐like regimen (23.5%).

These findings suggest that in real‐world settings, each TNT regimen carries a distinct profile of toxicity and disease control. Given the absence of definitive superiority after adjustment, the selection of a TNT regimen should be individualized—considering patient fitness, toxicity tolerance, institutional infrastructure, and surgical expertise.

### 4.3 Surgical Complexity

In the RAPIDO trial, operative time was similar between TNT and CRT groups (median 245 min in both arms), but blood loss was significantly higher in the TNT group (median 400 vs. 300 mL). Moreover, surgeons more often judged the quality of the mesorectal plane as suboptimal in TNT cases, despite comparable rates of R0 resection and CRM negativity [7, 40]. In contrast, findings from PRODIGE‐23 showed that perioperative morbidity and R0 resection rates were not worsened by TNT, and Clavien–Dindo≥grade 3 complications were slightly less frequent in the TNT group, although operative time and blood loss were not reported [8].

Beyond RCTs, real‐world data suggest that TNT carries regimen‐specific toxicity patterns and may introduce additional surgical challenges, such as tissue fibrosis and distorted anatomical planes. These issues may not always be fully captured in RCT endpoints but remain important considerations for clinical practice. Institutional audits have shown that extended intervals between radiation and surgery are linked to higher rates of circumferential margin positivity and incomplete TME [36]. A nationwide Austrian survey further confirmed that TNT was perceived to impair dissection quality (65%), plane identification (57%), and increase tissue fragility (47%) [37].

Taken together, while randomized trials generally suggest that TNT does not worsen perioperative safety, the RAPIDO findings and real‐world experiences highlight increased technical complexity, particularly in relation to dissection planes, and tissue handling.

A key limitation is the lack of direct comparisons between TNT and upfront surgery, which remains standard in Japan. Most trials compare TNT with CRT, leaving a gap in assessing TNT's safety and feasibility in the Japanese context. Japanese surgeons are less familiar with managing patients after preoperative radiotherapy, and the added burden of full‐course chemotherapy in TNT may increase surgical and perioperative challenges.

### 4.4 Residual Disease and the Hidden Risk of Distant Metastasis

A more nuanced concern in the TNT era is the behavior of residual disease—either in patients managed with NOM or in non‐responders to preoperative therapy.

In NOM, patients initially classified as super responders may later experience local regrowth. Recent studies have reported that local regrowth is associated with an increased risk of distant metastases [40, 41, 42, 43]. While regrowth rates vary, a large‐scale study from the International Watch & Wait Database (IWWD) reported that nearly 30% of patients developed local regrowth within 3 years. Importantly, patients with local regrowth had a significantly higher risk—nearly fivefold—of developing distant metastases compared to those without regrowth (HR 4.98; 95% CI, 3.20–7.78; *p* < 0.001) [40]. This finding supports the concern that delayed resection may permit systemic dissemination of treatment‐resistant tumor clones.

Non‐responders represent another concern. In these patients, TNT may fail to achieve adequate tumor regression, yet still delay curative surgery. This extended preoperative interval may allow tumor progression and increase metastatic potential, possibly explaining why survival gains seen in responders may be offset by poor outcomes in non‐responders.

These dual risks—regrowth after NOM and disease progression in non‐responders—highlight the current inability to reliably predict treatment response or residual tumor. This underscores the urgent need for biomarkers and early response evaluation tools to tailor TNT appropriately. Until such predictive methods become available, TNT should be applied with caution.

## 5 Guideline Positioning of TNT: Global Consensus and Divergences

### 5.1 Global Perspectives

Recent international guidelines increasingly acknowledge the role of TNT for LARC, yet their recommendations differ slightly in terms of target populations, preferred regimens, and emphasis on organ preservation (Table 3).

**TABLE 3:** Recommendations on TNT and NOM in recently updated international guidelines.

Guideline	TNT recommendation	TNT regimen	Position on NOM	Additional considerations
National Comprehensive Cancer Network (NCCN) (USA, 2024) [16]	Preferred approach for stage II/III rectal cancer	Induction chemotherapy with FOLFIRINOX is an option for T4, node‐positive rectal cancer.	Increasing in the USA, however, longer follow‐up, larger sample sizes, and additional careful observational studies are needed before cCR patients are routinely managed by NOM.	NOM may be considered in centers with experienced multidisciplinary teams after a careful discussion with the patient about their risk tolerance.
European Society for Medical Oncology (ESMO) (Europe, 2024) [17]	Recommended for high‐risk features (T4, MRF+, EMVI+, N2, lateral LN+) or when organ preservation is intended	The low local control rates in the RAPIDO trial and the lack of DFS benefit in the Polish II and STELLAR trials should be taken into account when considering TNT with SCRT.	Upfront CRT followed by consolidation chemotherapy might be the preferable sequence when NOM is intended.	NOM after TNT need shared‐decision making with the patient regarding the risk of regrowth after initial cCR, which necessitates salvage TME, and the need for strict monitoring schedule.
French Intergroup (France, 2025) [18]	Considered as the reference for T3‐T4 any N middle and low rectal adenocarcinoma.	FOLFIRINOX before CRT is the reference. SCRT followed by consolidation chemotherapy before surgery is not recommended.	Can be proposed in expert centers when cCR is strictly achieved, local resection is not possible, or the patient refuses radical surgery	The main problem of the NOM strategy is response evaluation after neoadjuvant therapy.
Japanese Society for Cancer of the Colon and Rectum (JSCCR) (Japan, 2024)	Weakly recommended against		Weakly recommended against	History of treatment advances centered on upfront surgery is a barrier to the introduction of the TNT and NOM concepts.

Abbreviations: CRT, chemoradiotherapy; DFS, disease‐free survival; EMVI, extramural venous invasion; MRF, mesorectal fascia; NOM, non‐operative management; SCRT, short‐course radiotherapy; TNT, total neoadjuvant therapy.

The NCCN Guidelines present the most inclusive stance, recommending TNT as a standard for all stage II/III rectal cancers. A range of protocols, including both induction and consolidation, is accepted, with emphasis on improved systemic control, pCR rates, and NOM feasibility [16].

The ESMO Guidelines take a more selective approach, reserving TNT for high‐risk cases (e.g., cT4, EMVI+, MRF+, N2). CRT or even upfront surgery remain valid options for lower‐risk patients. Notably, ESMO cautions against SCRT‐based consolidation regimens, citing local control issues in RAPIDO and absence of DFS benefit in Polish II and STELLAR [17].

The French Intergroup Guidelines align more closely with NCCN, recommending TNT for T3–T4, any N, middle/low rectal cancers. They endorse induction TNT with mFOLFIRINOX as the reference, while discouraging SCRT‐based consolidation due to concerns about surgical complications [18].

### 5.2 The Japanese Perspective: Conservative Endorsement in Context

The 2024 Japanese Colorectal Cancer Treatment Guidelines (Japanese version; English version in preparation) appear to contrast strikingly with the global trend. TNT and NOM are mentioned for the first time, but both are “weakly recommended against.” This cautious stance is the result of a combination of factors, including the unique history of treatment development, the limited domestic evidence available, and the unique healthcare system.

Historically, Japan did not incorporate preoperative radiation into standard rectal cancer management. Until the 2016 version, CRT was not endorsed at all [44], and it was only in the 2019 version that it was weakly recommended for cases where R0 resection was difficult [45]. Consequently, opportunities to consider NOM have been scarce, and clinical experience with non‐operative strategies is limited. It is likely that the concept of NOM—particularly the notion of omitting surgery in patients with a favorable response—will require considerable time to become accepted within Japanese surgical and patient communities.

Another key reason for Japan's cautious stance on TNT and NOM lies in the structure of its healthcare system. Rectal cancer surgery is performed not only in major centers but also in regional community hospitals, often by general surgeons with limited colorectal specialization. National guidelines are therefore designed not to restrict advanced treatment by specialists, but to provide a safe and consistent framework for a broader range of providers. In other words, the Japanese healthcare system prioritizes regional access, which, while promoting geographic equity, limits the feasibility of complex regimens like TNT or NOM. These approaches require coordinated multidisciplinary teams—including surgery, radiation oncology, and medical oncology—as well as access to intensive surveillance infrastructure. Such integrated care is often only achievable in high‐volume or specialized centers, highlighting the need for further centralization to enable safe implementation of TNT and NOM.

Patient characteristics also influence practice. Japanese patients generally have lower BMI and less visceral fat. This anatomical feature may increase the risk of radiation‐induced toxicity due to reduced adipose buffering of adjacent organs [46]. Japanese patients also tend to be diagnosed at earlier stages. As a result, oncological safe resection is often achievable without the need for preoperative downstaging, making upfront surgery a viable and appropriate option. Consequently, the relative advantages of TNT may be less pronounced in this population. In contrast, Western patients with obesity face higher surgical risks, making NOM more attractive.

Cultural factors are also significant. Japanese patients, compared to their Western counterparts, tend to prefer interventions that offer a clear sense of certainty and completeness. The concept of retaining tumor tissue during prolonged preoperative therapy, or undergoing active surveillance without surgery, may cause psychological distress. Even if NOM avoids a permanent stoma, fear of regrowth may outweigh this benefit.

## 6 Research Directions in Japan

### 6.1 Evidence Gap in Japanese Practice

A key issue is the absence of robust clinical data tailored to Japanese practice. Most evidence supporting TNT originates from Western populations, where patient characteristics, surgical techniques, and healthcare systems differ substantially. In Japan, upfront surgery without preoperative CRT remains the standard of care, and therefore it is not feasible to directly extrapolate the situation in other countries to Japan, particularly with regard to toxicity and the feasibility of surgery.

### 6.2 Urgent Need for Contextualized Prospective Trials

To address these concerns, prospective trials tailored to the Japanese setting are urgently needed. Most existing data come from small, single‐center studies.

ENSEMBLE‐1 was the first multicenter phase II trial in Japan to evaluate TNT, using SCRT followed by CAPOX and then TME or NOM. It showed a 30% pCR rate and a combined pCR + cCR rate of 43.3%, with no treatment‐related deaths [47]. ENSEMBLE‐2 subsequently examined CRT followed by CAPOX; among 28 patients, pCR was 23.8%, pCR + sustained cCR reached 35.7%, and NOM was feasible in 21.4%, with high protocol completion [48].

The ongoing phase III ENSEMBLE trial compares SCRT + CAPOX vs. CAPOXIRI in cStage II–III low rectal cancer. The primary endpoint is organ preservation–adapted DFS [49]. In parallel, JCOG2207 (MARIAGE) compares standard TME + LPLND to TNT with SCRT and CAPOX ± selective LPLND, with OS as the primary endpoint.

These trials aim to clarify the role of TNT in Japan, not only in terms of oncologic outcomes, but also surgical complexity, postoperative morbidity, quality of life, and cost‐effectiveness. Reliable cCR assessment must also be developed to enable safe NOM adoption.

### 6.3 Alternative Sequencing Strategies in TNT

While both ENSEMBLE and MARIAGE adopt consolidation‐based TNT, the latest research findings suggest that, in cases where NOM is not intended and surgery is anticipated, a sequence based on induction therapy may be a better option [50].

Furthermore, recent French trials propose a selective TNT approach—starting with chemotherapy and adding radiotherapy only for poor responders [24, 25]. Such biologically adapted models may be especially relevant in Japan, where CRT is not routinely used, given the importance of balancing efficacy and tolerability.

## 7 Conclusion

TNT has transformed rectal cancer treatment by enhancing tumor response, reducing metastases, and enabling NOM in selected cases. Phase III trials consistently show improved pCR and systemic control, challenging conventional sequencing. However, concerns remain regarding treatment burden, toxicity, delayed surgery in non‐responders, and unclear OS benefit. Its implementation demands multidisciplinary coordination and infrastructure—often lacking in general practice.

Reflecting these challenges, the 2024 Japanese guidelines adopt a cautious stance, citing limited domestic data and healthcare system constraints. TNT should not be a universal standard but a context‐dependent strategy. In Japan, its optimal use will require individualized care, centralization, and evidence tailored to local practice—balancing oncologic benefit with quality of life.

## Author Contributions

**Kay Uehara:** conceptualization, writing – original draft, investigation. **Akihisa Matsuda:** methodology, formal analysis, writing – review and editing. **Takeshi Yamada:** writing – review and editing, supervision. **Aitsariya Monkhonsupphawan:** writing – review and editing. **Hiroshi Yoshida:** supervision, writing – review and editing.

## Ethics Statement

The authors have nothing to report.

## Consent

The authors have nothing to report.

## Conflicts of Interest

Author K.U. is an editorial board member of the Annals of Gastroenterological Surgery. The other authors have no competing interests to declare that are relevant to the content of this article.
